# Novel block glycopolymers prepared as delivery nanocarriers for controlled release of bortezomib

**DOI:** 10.1007/s00396-018-4406-8

**Published:** 2018-09-24

**Authors:** Xiaoting Zhang, Tianyu Yuan, Hailiang Dong, Jiaming Xu, Danyue Wang, Han Tong, Xiaohuan Ji, Bin Sun, Meifang Zhu, Xiaoze Jiang

**Affiliations:** State Key Laboratory for Modification of Chemical Fibers and Polymer Materials, College of Materials Science and Engineering, Donghua University, Shanghai, 201620 People’s Republic of China

**Keywords:** Block glycopolymers, Bortezomib, Dynamic covalent bonding, Self-assembly, Controlled release

## Abstract

To explore block glycopolymers as novel polymeric delivery nanocarriers for anticancer drug bortezomib (BTZ), three types of block glycopolymers, poly(ethylene glycol)-*block*-poly(gluconamido ethyl methacrylate) (PEG_113_-*b*-PGAMA_20_), poly(ethylene glycol)-*block*-poly(styrene)-*block*-poly(gluconamido ethyl methacrylate) (PEG_113_-*b*-PS_50_-*b*-PGAMA_20_), and poly(ethylene glycol)-*block*-poly(2-(diethyl amino) ethyl methacrylate)-*block*-poly(gluconamido ethyl methacrylate) (PEG_113_-*b*-PDEA_50_-*b*-PGAMA_20_), were synthesized via atom transfer radical polymerization (ATRP) using a PEG-based ATRP macroinitiator. Three glycopolymers possess the capacity to load BTZ via pH-induced dynamic covalent bonding and/or hydrophobic interaction with their specific self-assembly behaviors, and PEG_113_-*b*-PS_50_-*b*-PGAMA_20_ carrier maintains the sustain release behavior of BTZ due to the stable micellar structure; PEG_113_-*b*-PDEA_50_-*b*-PGAMA_20_ carrier realizes the abrupt release at pH 5.5 by collapse of micellar structure, while PEG_113_-*b*-PGAMA_20_ carrier exhibits the fastest release at studied solution pHs. This study would provide a light to develop novel block glycopolymer carrier for the delivery of anticancer drug bearing boronic acid groups.

**Graphical abstract:**
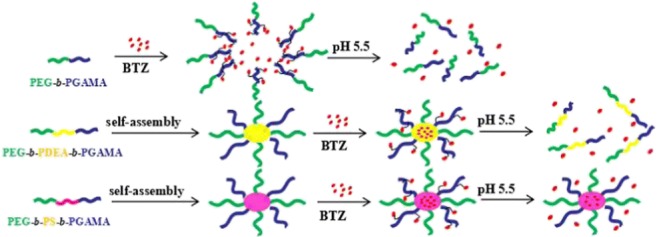
ᅟᅟ

ᅟᅟ

## Introduction

Block copolymers and their self-assemblies have been drawn more attention for their potential applications on the drug delivery fields due to the capacity of self-assembling spontaneously into micellar aggregates with core-shell structure and the size at a nanometer scale to control drug loading and release behaviors [1–5].

Block copolymers after self-assembly in water media were originally developed to be delivery systems from amphiphilic block copolymers, and then extend to double hydrophilic block copolymers (DHBCs) by eliminating the usage of organic solvents and importantly endowing the stimuli-responsiveness of polymeric carriers [6–12]. Among studied DHBCs, pH-sensitive block copolymers were extensively utilized as polymeric carriers to achieve drug stability at physiological pH of 7.4 and pH-triggered release of anticancer drug only at the slight acidic pH by mimicking the pH conditions of normal tissue and tumor tissue [13–16].

The drug loading strategy for copolymer carriers was explored conventionally from physical interactions between the cores of copolymer carriers with hydrophobic drugs, then further developed to chemical conjugations of drug to copolymer carriers via pH-induced degradation or cleavage bonding [17–20]. The strategy of physical interactions provides the passive weak affinity by convenient preparation process then inevitably causes the premature release of loaded drug before arriving the target position. Chemical conjugation methodology via characteristic fragile covalent bonds guaranteed the stability of drug-loaded polymeric carriers during transportation and controlled release of drug after reached target positions, while the systems used were generally designed and prepared via complicated and multi-step rigorous organic synthesis [17–20].

Dynamic covalent bonding has been considered recently as an alternative strategy for the encapsulation and release of anticancer drug, which meets the fundamental principles of smart carriers for requirements of stability during transportation and spontaneous and controlled release only in cancer cell after administration via stimuli-responsiveness without rigorous preparation process [21–24]. Among the studied dynamic covalent bonds, the dynamic interaction between the boronic acid and 1, 2-diols or 1, 3-diols was recently introduced to copolymer carriers for drug delivery field due to the well-known pH-induced dynamic and reversible interactions, which complexes to form strong covalent bond of boronate esters at basic conditions then spontaneously breaks via reversible dissociation at mildly acidic pH solutions, and their reversible pH interaction region matched closely with pH differentiation of normal cells and tumor cells [25–28].

Following the strategy of dynamic covalent bonding, Messersmith [29] and co-workers designed and prepared the PEGylated polymers containing catechol moiety on the polymer chain terminal to exploit the loading and release behavior of the anticancer drug, bortezomib (BTZ), which was a dipeptide boronic acid analogue and investigated to be a novel class of potent and effective antitumor agents, and the results confirmed that these ortho-diols containing polymers are able to dynamically conjugate with BTZ at physiological pH of 7.4 and spontaneously dissociate from BTZ at pH 5.5 close to tumor environment. On the base of boronic acid-diols interaction, different systems including dual-responsive boronate cross-linked micelles or reversible boronate cross-linked nanocarriers have been already developed in target drug delivery field [30, 31]. Those studies pioneer that the polymer carrier with catechol or borate-containing moieties have the ability to bind and release borate-containing or other therapeutics in a pH-dependent manner, while, catechol was only focused and complicatedly introduced to studied systems and the capacity of boronic acid-diols interaction was much limited by the diols numbers of used polymers.

Glycopolymers were developed recently by Narain and Armes group [32, 33] due to the following characteristics: hydrophilicity, biocompatibility, and functionality of abundant hydroxyl groups, and proofed pH-induced dynamic reversible boronic acid-diols interaction between glycopolymers with borate-containing polymers. Appelhans [34] and co-workers further studied and reported that dendritic glycopolymer was used as drug delivery system for BTZ in bone cements for locally treating osteolytic bone lesions in a short-term retarded release manner. Therefore, it is necessary to develop novel block glycopolymers with well-defined compositions and structures used as drug delivery systems to investigate the loading and release behavior of BTZ via glycopolymer-boronic acids interactions.

Herein, one diblock glycopolymer, poly(ethylene glycol)-*b*-poly(gluconamido ethyl methacrylate) (PEG-*b*-PGAMA), and two triblock glycopolymers, poly(ethylene glycol)-*block*-poly(styrene)-*block*-poly(gluconamido ethyl methacrylate) (PEG-*b*-PS-*b*-PGAMA), and poly(ethylene glycol)-*block*-poly(2-(diethylamino)ethyl methacrylate)-*block*-poly(gluconamido ethyl methacrylate) (PEG-*b*-PDEA-*b*-PGAMA), were synthesized via atom transfer radical polymerization (ATRP) with no middle block, non-responsive segment PS middle block, and pH-responsive segment PDEA middle block with the same DPs. Those glycopolymers were then utilized as drug delivery system to investigate the loading and release behaviors of BTZ.

## Experimental

### Materials

Monohydroxy-capped poly(ethylene glycol) (PEG_113_-OH) with a mean degree of polymerization, DP, of 113 (*M*_n_ = 5000 g/mol, *M*_w_/*M*_n_ = 1.02) was purchased from Aldrich and dried at 30 °C under vacuum overnight prior to use. 2-Diethylaminoethyl methacrylate (DEA, Aldrich, 99%) and styrene (St, Aldrich, 99%) were passed through basic alumina column to remove inhibitor and then vacuum distilled from CaH_2_ and stored at − 20 °C prior to use. 2, 2-Bipyridine (bpy), copper bromide (CuBr), 2-bromoisobutyryl bromide, 2-aminoethyl methacrylate hydrochloride (AMA^.^HCl), d-glucono-δ-lactone, bortezomib (BTZ), and Alizarin Red S (ARS) were all purchased from J&K and directly used. Methanol, toluene, triethylamine (TEA), 1-methyl-2-pyrrolidinone (NMP), and isopropyl alcohol (IPA) were purchased from Sinopharm Group Chemical Reagent Co., Ltd., and then distilled from CaH_2_ under reduced pressure. Dichloromethane, chloroform, dimethyl sulfoxide (DMSO), and other organic solvents were purchased from Sinopharm Group Chemical Reagent Co., Ltd., and directly used without further purification. Bromide terminated poly(ethylene glycol) (PEG_113_-Br) macroinitiator and 2-gluconamidoethyl methacrylate (GAMA) monomer were prepared from PEG_113_-OH and 2-bromoisobutyryl bromide, and AMA^.^HCl and d-glucono-δ-lactone, respectively, according to literature procedures [35, 36].

### Preparation of PEG-*b*-PGAMA diblock copolymer

A reaction flask with a magnetic stirrer and a rubber septum was charged with PEG_113_-Br macroinitiator (0.25 g, 0.049 mmol), GAMA (0.30 g, 0.98 mmol), bpy (0.015 g, 0.098 mmol), and NMP (2.0 mL). The reaction mixture was purged N_2_ atmosphere for 30 mins, then CuBr (0.007 g, 0.049 mmol) was added to the reaction flask under a nitrogen atmosphere to start the polymerization at 40 °C. After 4 h, The reaction mixture was dialyzed against distilled water for 2 days to remove copper catalysts and residual monomer and followed by lyophilization to obtain the white solid (0.45 g, yield 82%).

### Preparation of PEG-*b*-PS-Br diblock copolymer

A reaction flask with a magnetic stirrer and a rubber septum was charged with PEG_113_-Br macroinitiator (1.0 g, 0.2 mmol), St (1.5 mL, 11 mmol), CuBr (0.029 g, 0.2 mmol), and bpy (0.063 g, 0.4 mmol). The flask was degassed by three freeze-pump-thaw cycles, backfilled with N_2_, and then placed in an oil bath at 100 °C to start the polymerization. After 4 h, the flask was quenched in liquid nitrogen, exposed to air, and then diluted with 15 mL of chloroform. After passing through a silica gel column to remove the copper catalysts, the solvent was concentrated and precipitated into excess n-hexane by repeating three times, the obtained solid was then dried in a vacuum oven overnight at room temperature (1.2 g, yield 56%).

### Preparation of PEG-*b*-PS-*b*-PGAMA triblock copolymer

A reaction flask with a magnetic stirrer and a rubber septum was charged with PEG_113_-*b*-PS_50_-Br (0.5 g, 0.05 mmol), GAMA (0.46 g, 1.5 mmol), bpy (0.008 g, 0.05 mmol), and NMP (2.0 mL). The reaction mixture was purged N_2_ atmosphere for 30 mins, then CuBr (0.014 g, 0.1 mmol) was added to the reaction flask under a nitrogen atmosphere to start the polymerization at 40 °C. After 15 h, The reaction mixture was dialyzed against distilled water using a dialysis membrane (Molecular weight Cut Off (MWCO) 3500) for 2 days to remove copper catalysts and residual monomer and followed by lyophilization to obtain the white solid (0.7 g, yield 73%).

### Preparation of PEG-*b*-PDEA-*b*-PGAMA triblock copolymer

A reaction flask with a magnetic stirrer and a rubber septum was charged with PEG_113_-Br macroinitiator (0.25 g, 0.048 mmol), DEA (0.38 g, 2.4 mmol), bpy (0.015 g, mmol), and NMP (2.0 mL). The reaction mixture was purged N_2_ atmosphere for 30 mins, then CuBr (0.007 g, 0.048 mmol) was added to the reaction flask under a nitrogen atmosphere to start the polymerization at 40 °C. After 6 h, the conversion of DEA reaches above 95% as judged by ^1^H NMR at time intervals. An aliquot of reaction solution was withdrawn for subsequent gel permeation chromatography (GPC) analysis before the introduction of a degassed mixture of GAMA (0.44 g, 1.44 mmol) and NMP (2.0 mL) via a double-tipped needle. After 24 h, the reaction was quenched in liquid nitrogen, exposed to air, and dialyzed against distilled water using a dialysis membrane (MWCO 3500) for 2 days to remove copper catalysts and residual monomer and followed by lyophilization to obtain the white solid (0.86 g, yield 80%).

### Preparation of micelles

The obtained PEG-*b*-PS-*b*-PGAMA glycopolymer (40 mg) was dissolved completely in 2 mL of DMSO overnight, then 10 mL of diluted NaOH aqueous solution (pH 9) was slowly added dropwise under stirring. The solution was then dialyzed against distilled water using a dialysis membrane (MWCO 3500) for 24 h and then finally freeze-dried to give glycopolymer micelles. Forty milligrams of the obtained PEG-*b*-PDEA-*b*-PGAMA glycopolymer was dissolved molecularly in 10 mL diluted HCl aqueous solution (pH 3) overnight, and self-assembled into micelles at pH 9 by addition of diluted NaOH aqueous solution, and then freeze-dried to give glycopolymer micelles for measurements.

### Preparation of drug-loaded micelles

The typical procedure followed the method reported [37], 4 mg of BTZ and 40 mg of glycopolymers were accurately weighted to dissolve in 2 mL of DMSO, then 10 mL of diluted NaOH aqueous solution (pH 9) was slowly added dropwise under stirring. The solution was dialyzed against distilled water using a dialysis membrane (MWCO 3500) for 24 h, and then freeze-dried to give drug-loaded glycopolymer micelles.

### Quantification of the amount of BTZ loaded into the glycopolymers

A series of solutions of BTZ with different concentrations in DMSO were prepared, and the absorbance at 269 nm was measured by UV-Vis spectrophotometer to build the standard ultraviolet absorption curve of BTZ in DMSO, which was used for the calculation of entrapment efficiency (EE%) and loading capacity (LC%) of BTZ loaded by glycopolymers. In the same way, the standard ultraviolet absorption curves of BTZ were obtained in phosphate buffer (pH 7.4, 0.01 M) and acetate buffer (pH 5.5, 0.01 M), respectively.

### Release of BTZ from glycopolymers in vitro

A solution (5 mL) of BTZ-loaded glycopolymer (BTZ concentration, 1.0 mg/mL) in phosphate buffer (pH 7.4,, 0.01 M) was dialyzed in a dialysis membrane (MWCO 3500) against 40 mL of acetate buffer (pH 5.5, 0.01 M) or phosphate buffer (pH 7.4, 0.01 M) at 37 °C and 100 rpm. After time intervals, 5 mL of buffer solution outside the dialysis membrane was taken out for determining the amount of BTZ by measuring the absorbance at 269 nm using UV-Vis spectrophotometer, meanwhile, 5 mL of fresh buffer solution was added.

## Characterizations

### ^1^H NMR and GPC

All ^1^H NMR spectra were recorded in D_2_O, CDCl_3_, or deuterated DMSO using a Bruker 400 MHz spectrometer. Molecular weight and molecular weight distribution of glycopolymers were assessed by gel permeation chromatography (GPC) in DMF containing 0.5 g/L LiBr at a flow rate of 1.0 mL min^−1^ using a series of three linear Styragel columns (HT2, HT4, and HT5) or two linear Styragel columns (HT4 and HT5), a Waters 1515 pump and waters 2414 differential refractive index detector (set at 30 °C) and an oven temperature of 60 °C. Calibration was performed using a series of near-monodisperse polystyrene standards.

### DLS and TEM

The size and size distribution of copolymer micelles were measured using the Zetasizer (Malvern Nano ZS). The samples were prepared at 1 mg/mL concentration, and removed dust by filtering using a 0.45 μm filter membrane before measurement. The size and size distribution of micelles were recorded by tested samples via 15 runs and each run keeps 10 s, every sample was measured three times and the size was averaged. The scattering angle is 173° for presented size distribution data. Transmission electron microscopy (TEM) images were recorded using a Tecnai G220 TWIN electron microscope at an accelerating voltage of 1 kV. TEM samples were prepared by placing dilute aqueous solutions (0.1 g/L, pH 9) of polymeric micelles on copper grids coated with thin films of Formvar and carbon. No staining was required.

### Fluorescence

Critical micelle concentrations of block glycopolymers were analyzed by PerkinElmer Lambda Fluorescence Spectrometer with Nile red as a probe and 550 nm of excitation wavelength to record emission spectra of samples in the range from 560 to 600 nm.

### UV-Vis

The standard UV-visible absorption curve of BTZ, entrapment efficiency of drug, and cumulative drug-releasing curves were measured by UV-Vis spectrophotometer (Beijing General Analytical Spectrometer). The release of BTZ from glycopolymer nanocarriers was manipulated on Constant Temperature Shaker TS-100C to support continuous shock when dialysis membranes containing drug-loaded carriers were put on PBS buffer solution of pH 7.4 or pH 5.5.

## Results and discussions

### Synthesis of block glycopolymers

**Scheme 1:**
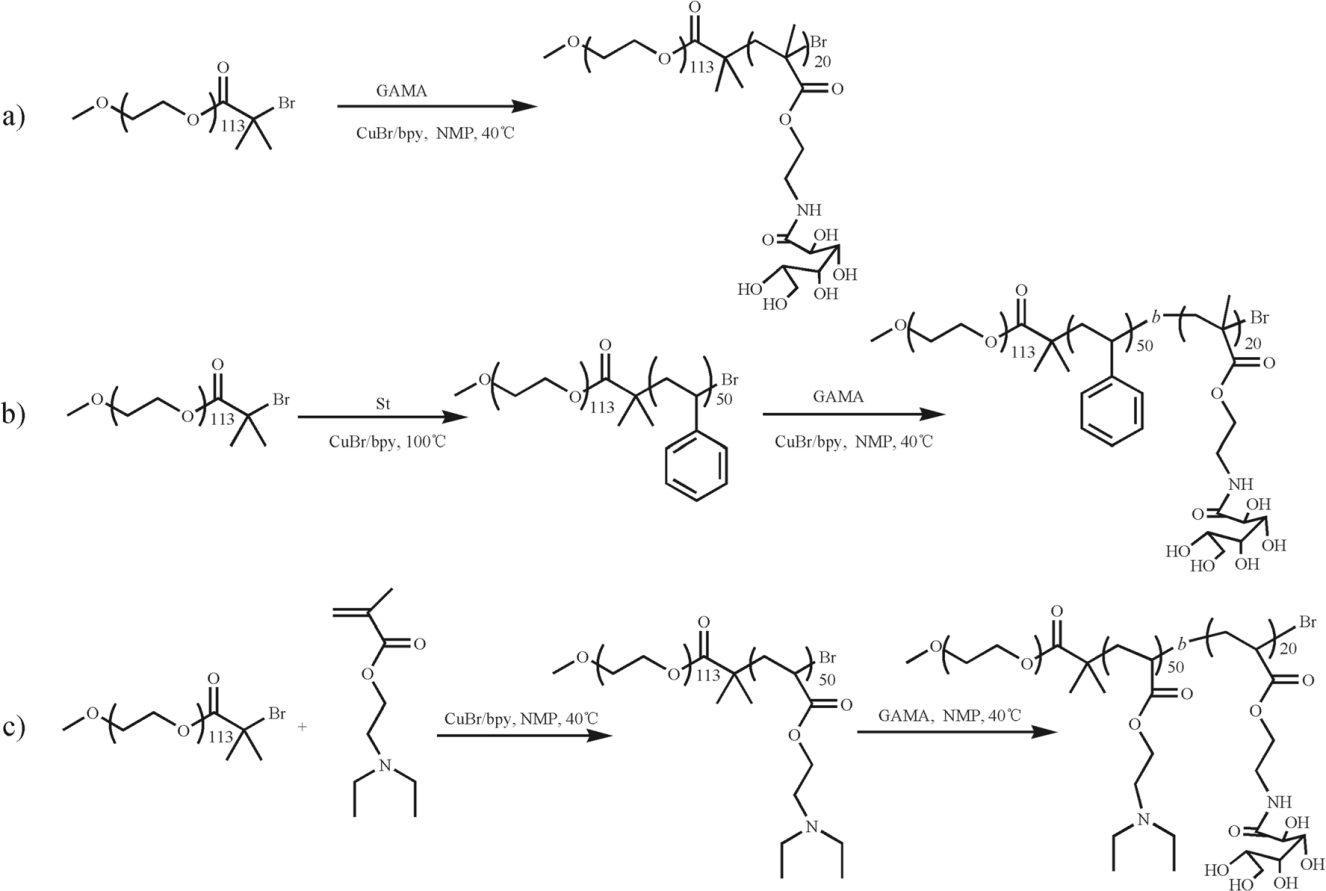
Synthetic routes employed for the preparation of block glycopolymers

With the development of controlled/living radical polymerization, different glycopolymers with well-defined structures and controlled components had already been developed via atom transfer radical polymerization (ATRP) [38, 39] especially without protection chemistry method [32, 33]. To verify the pH-induced interaction of glycopolymers with boronic acid, poly(ethylene glycol)-*block*-poly(gluconamido ethyl methacrylate) (PEG-*b*-PGAMA) diblock glycopolymer was prepared via ATRP technique by using PEG-Br as the macroinitiator and CuBr/bpy as the catalysts at 40 °C as shown in Scheme 1. The polymerization solvent was chose to be 1-methyl-2-pyrrolidinone (NMP) instead of protonic alcohol for better solvent solubility to GAMA and lower volatility under N_2_ atmosphere.

After polymerized 4 h, the conversion of GAMA had reached more than 95% as judged by ^1^H NMR, and signals of both blocks of diblock glycopolymer after purified by lyophilization were observed in ^1^H NMR spectrum in D_2_O, and the degree polymerization (DP) of GAMA was calculated to be 20 based on the integral ratio of characteristic peaks of GAMA at δ 4.0–4.3 ppm with that of PEG at δ 3.6 ppm. GPC trace of diblock glycopolymer reveals a monomodal and symmetric peak with an *M*_n_ of 6150 and *M*_w_/*M*_n_ of 1.20. Then the obtained glycopolymer was denoted as PEG_113_-*b*-PGAMA_20_.

To check effect of hydrophobic or pH-responsive components of glycopolymers used as drug delivery systems on the loading and release behaviors of boronic acid-containing BTZ, polystyrene (PS) or poly(2-(diethylamino)ethyl methacrylate) (PDEA) were introduced to the middle block of PEGylated glycopolymer via conventional ATRP chain extension method as shown in Scheme 1.

**Fig. 1:**
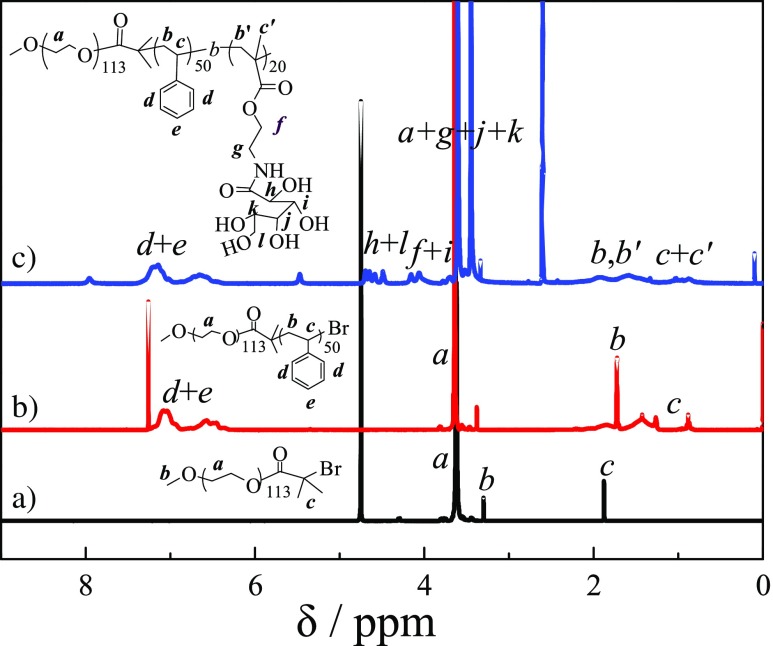
^1^H NMR spectra recorded for (*a*) PEG_113_-Br macroinitiator in D_2_O, (*b*) PEG_113_-*b*-PS_50_-Br precursor in CDCl_3_, and (*c*) PEG_113_-*b*-PS_50_-*b*-PGAMA_20_ triblock glycopolymer in *d*_6_-DMSO

**Fig. 2:**
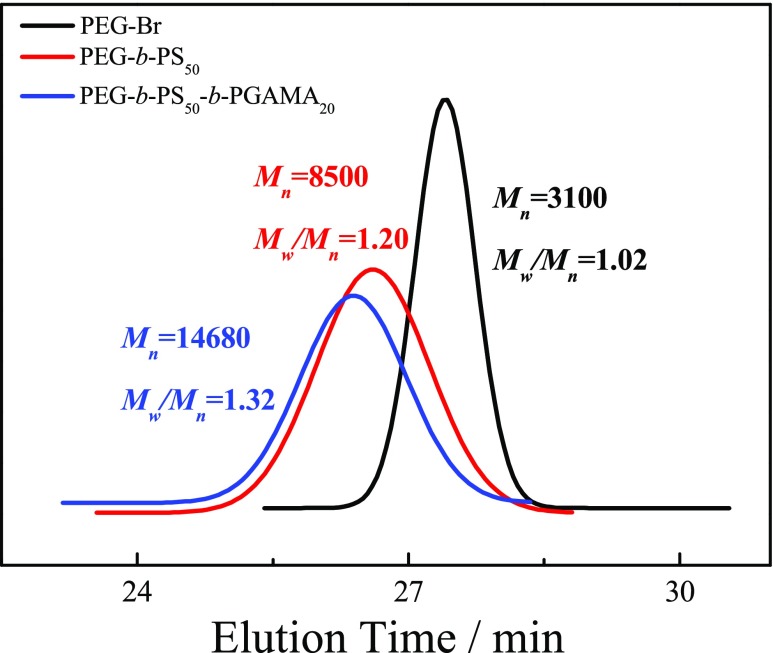
GPC traces in DMF eluent for PEG_113_-Br macroinitiator, PEG_113_-*b*-PS_50_-Br precursor, and PEG_113_-*b*-PS_50_-*b*-PGAMA_20_ triblock glycopolymer

PEG-Br was used as macroinitiator to polymerize the styrene monomer with CuBr/bpy as the catalysts at 100 °C for 4 h to obtained PEG-*b*-PS-Br diblock copolymer. The DP of PS was calculated to be 50 from ^1^H NMR spectrum of PEG-*b*-PS in CDCl_3_ (shown in Fig. 1b) by comparing the peak integrals of the benzene proton signal of PS at δ 6.5–7.0 ppm to that of two methylene groups of PEG at δ 3.6 ppm, and GPC trace of PEG-*b*-PS revealed a monomodal and symmetric peak with an *M*_n_ of 8150 and *M*_w_/*M*_n_ of 1.20 in Fig. 2, then PEG_113_-*b*-PS_50_-Br diblock copolymer precursor was used to polymerize GAMA monomer in NMP at 40 °C for 24 h via chain extend polymerization. The DP of GAMA was measured by ^1^H NMR spectrum of PEG-*b*-PS-*b*-PGAMA in *d*_6_-DMSO (shown in Fig. 1c) to be 20 by calculating the integral ratio of characteristic peaks of GAMA at δ 4.0–4.3 ppm with that of PS at δ 6.5–7.3 ppm. The monomodal and symmetric GPC peak was also observed for PEG-*b*-PS-*b*-PGAMA triblock copolymer, most importantly, there is a clear shift to a higher molecular weight for triblock copolymer in comparison to that of diblock copolymer, GPC analysis as shown in Fig. 2 revealed an *M*_n_ of 14,680 and *M*_w_/*M*_n_ of 1.32. Thus, the obtained triblock polymer was denoted as PEG_113_-*b*-PS_50_-*b*-PGAMA_20_ triblock glycopolymer.

**Fig. 3:**
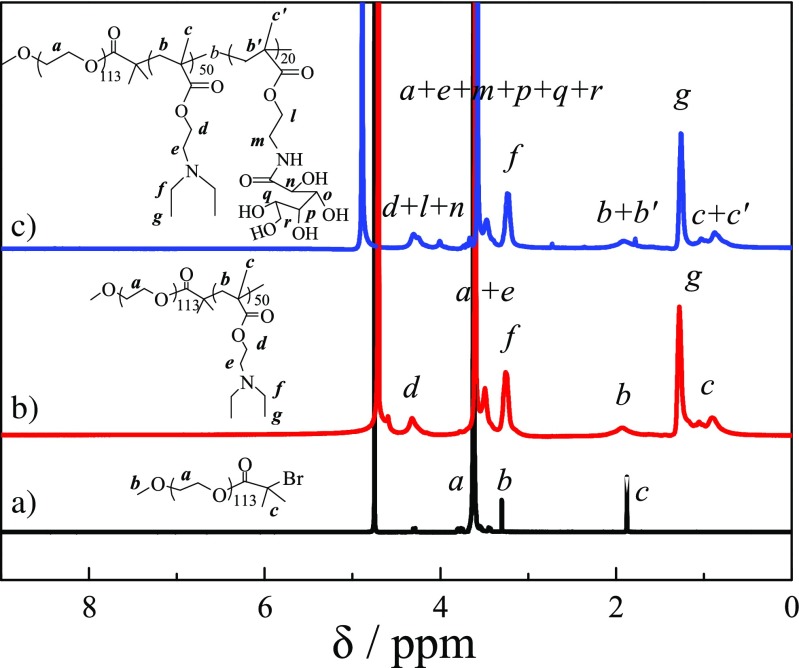
^1^H NMR spectra recorded for (a) PEG_113_-Br macroinitiator, (c) PEG_113_-*b*-PDEA_50_-Br precursor in D_2_O at pH 5, and (c) PEG_113_-*b*-PDEA_50_-*b*-PGAMA_20_ triblock glycopolymer in D_2_O at pH 5

**Fig. 4:**
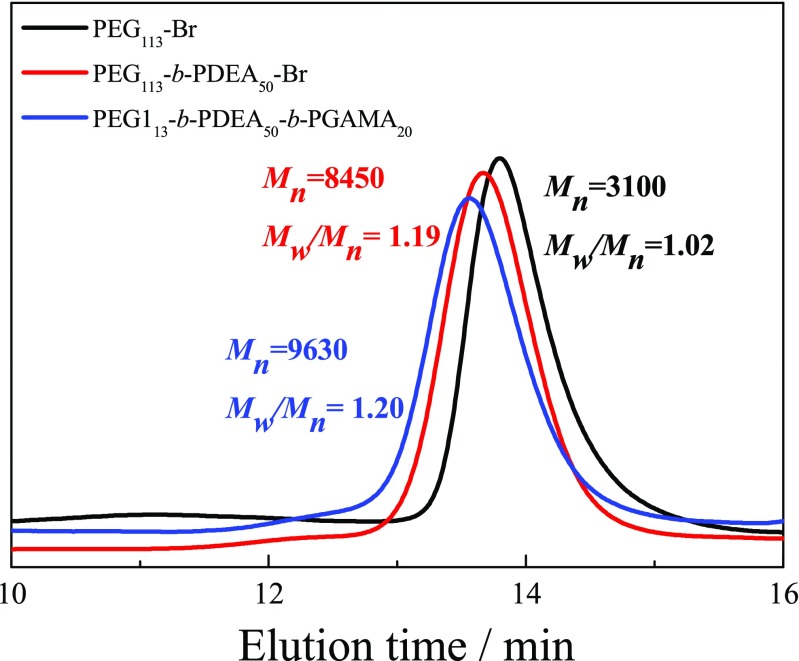
GPC traces in DMF eluent for PEG_113_-Br macroinitiator, PEG_113_-*b*-PDEA_50_-Br precursor, and PEG_113_-*b*-PDEA_50_-*b*-PGAMA_20_ triblock glycopolymer

Poly(ethylene glycol)-*block*-poly(2-(diethylamino)ethyl methacrylate)-*block*-poly-(gluconamido ethyl methacrylate) (PEG-*b*-PDEA-*b*-PGAMA) was synthesized from PEG-Br by successively polymerized PDEA monomer and then GAMA monomer in NMP at 40 °C by controlling the conversion of DEA monomer reached above 95% before the addition of GAMA monomer. After polymerization, the DP of PDEA and PGAMA were measured to be 50 and 20 by calculating the integral ratio of characteristic peaks of PDEA at δ 3.2 ppm and PGAMA at δ 4.0–4.3 ppm with that of PEG at δ 3.6 ppm (as shown in Fig. 3). Both GPC traces in Fig. 4 revealed the monomodal and symmetric peaks with *M*_n_ of 8450 and *M*_w_/*M*_n_ of 1.19, and *M*_n_ of 9630 and *M*_w_/*M*_n_ of 1.20 for PEG-*b*-PDEA precursor and PEG-*b*-PDEA-*b*-PGAMA triblock glycopolymer, respectively. Thus, the resultant copolymer was denoted as PEG_113_-*b*-PDEA_50_-*b*-PGAMA_20_ triblock glycopolymer. It should be noted that the object of this study mainly investigated the influence of glycopolymer and micelles with stable or pH-responsive cores prepared from triblock glycopolymers on the loading and release behaviors of BTZ. Therefore, two aspects should be considered to design those glycopolymers, degree polymerization (DP) of PGAMA block of glycopolymers should be controlled to be able to bind BTZ at pH 7.4, and triblock glycopolymers could form the stable micelles at pH 7.4 and exhibited the loading and release behaviors of BTZ, which were mainly controlled by the core properties of triblock glycopolymer micelles. Herein, based on our preliminary experiments, the DPs of PGAMA and PDEA (or PS) were fixed to be 20 and 50, respectively, PEG_113_-*b*-PGAMA_20_ diblock glycopolymer, PEG_113_-*b*-PS_50_-*b*-PGAMA_20_, and PEG_113_-*b*-PDEA_50_-*b*-PGAMA_20_ triblock glycopolymer were thus prepared via ATRP method.

### Self-assembly behaviors of block glycopolymers into micelles

Those block glycopolymers were designed for drug delivery systems and required to possess the basic capability of loading anticancer drug by forming the micellar nano-structures with core-shell type in water media or/and complexed with drugs via specific interactions.

Both PEG and PGAMA blocks exhibit well water solubility at the entire studied pH ranges. PS block is a typical hydrophobic segment without any stimuli-responsiveness, whereas PDEA block has been intensively studied as typical pH-responsive block and confirmed also to be a weak polybase with a *p*K_a_ of 7.3, which is water-insoluble generally to form micellar nanoaggregates at neutral or alkaline pHs and molecularly soluble as a weak cationic polyelectrolyte with unimer state at acidic pH attributed to protonation of its tertiary amine groups [40–42].

On the entire pH ranges, the dilute aqueous solution of PEG_113_-*b*-PGAMA_20_ was clear without typical Tyndall effect, and the presence of all signals of each block of PEG_113_-*b*-PGAMA_20_ could be observed and no any signals changed with solution pH changes in NMR spectra, indicating the PEG_113_-*b*-PGAMA_20_ block glycopolymer molecularly dissolves in water media with unimer state at studied solution pHs.

**Fig. 5:**
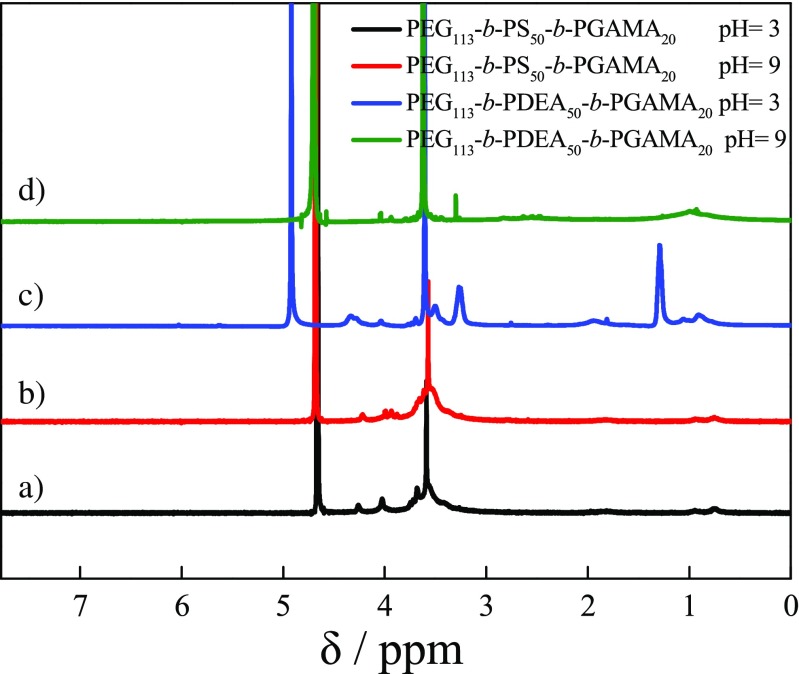
^1^H NMR spectra recorded in D_2_O for PEG_113_-*b*-PS_50_-*b*-PGAMA_20_ triblock glycopolymer at pH 3 (a) and pH 9 (b) and PEG_113_-*b*-PDEA_50_-*b*-PGAMA_20_ triblock glycopolymer at pH 3 (c) and pH 9 (d)

The dilute aqueous solution of PEG_113_-*b*-PS_50_-*b*-PGAMA_20_ triblock glycopolymer was bluish tinge, and the Tyndall effect could be clearly observed, which exhibits the distinguished feature of micellar solutions. The ^1^H NMR spectra recorded for the PEG_113_-*b*-PS_50_-*b*-PGAMA_20_ triblock glycopolymer at pH 3 and 9 as shown in Fig. 5a, b exhibit the presence of all signals for the PEG and PGAMA blocks and were visible, whereas the characteristic signals of benzene ring of PS block at δ 6.5–7.4 ppm disappear at both solution pHs, indicating that PEG_113_-*b*-PS_50_-*b*-PGAMA_20_ triblock glycopolymer self-assembled into micelles with core of hydrophobic PS block and shell of hydrophilic PEG and PGAMA blocks both at acidic and alkaline pH.

The dilute aqueous solution of PEG_113_-*b*-PDEA_50_-*b*-PGAMA_20_ triblock copolymer displayed bluish tinge above pH 8. ^1^H NMR spectra (shown in Fig. 5c, d) were recorded for the PEG_113_-*b*-PDEA_50_-*b*-PGAMA_20_ triblock copolymer at pH 3 and 9, respectively. All the signals of each block were visible including the characteristic signals of the PDEA block at δ 1.3 ppm and δ 3.23 ppm at pH 3, while those two signals of the PDEA block totally disappeared when pH changed to pH 9 with no any changes of all signals of the PEG and PGAMA blocks, indicating the pH-induced formation of the PDEA-core micelles with a shell consisting of the water-soluble PGAMA and PEG at alkaline pH, then dissociated to unimers spontaneously and reversibly at acidic pH solution.

**Fig. 6:**
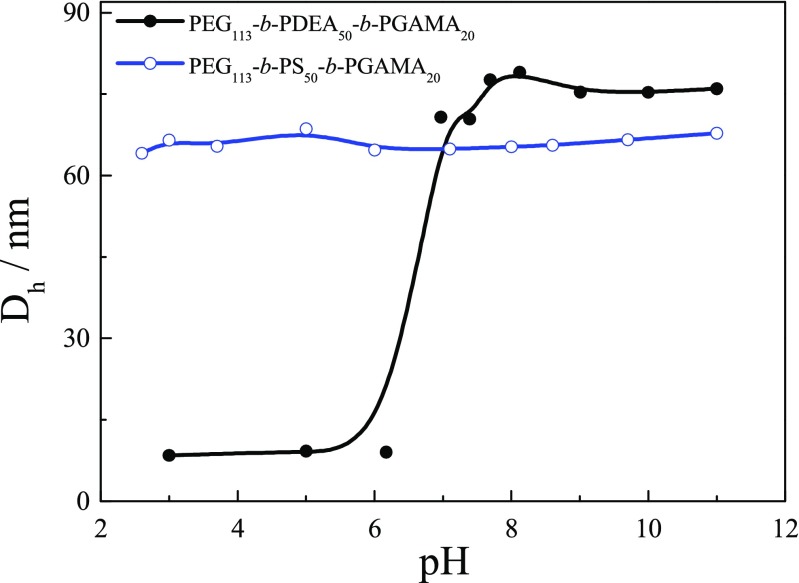
pH dependent manner of the mean hydrodynamic diameter ⟨*D*_h_⟩ for 1 g/L aqueous solutions of PEG_113_-*b*-PS_50_-*b*-PGAMA_20_ and PEG_113_-*b*-PDEA_50_-*b*-PGAMA_20_ triblock glycopolymers.

Those self-assembled behaviors of block glycopolymer were further investigated by dynamic light scattering (DLS) as shown in Fig. 6. The measured results show that micelles prepared from PEG_113_-*b*-PS_50_-*b*-PGAMA_20_ triblock glycopolymer were obtained with average hydrodynamic diameter ⟨*D*_h_⟩ of 60 nm and the size of micelles keeps constant and almost no change at studied pH range, which further confirms that the PS-core micelles prepared from PEG_113_-*b*-PS_50_-*b*- PGAMA_20_ triblock glycopolymer were stable at water media without pH-responsive property. There is no micellar behavior of PEG_113_-*b*-PDEA_50_-*b*-PGAMA_20_ triblock glycopolymer at acidic pH (blow pH 6) solution with low light scattering intensity, while it self-assembles into micelles with ⟨*D*_h_⟩ of 70 nm when pH is above 7 with strong light scattering intensity, indicating the formation of PDEA-core micelles at neutral and alkaline pH solution. It should be noted that PEG_113_-*b*-PGAMA_20_ triblock glycopolymer exists in unimers with average hydrodynamic diameter ⟨*D*_h_⟩ of 9 nm at studied pH range.

**Fig. 7:**
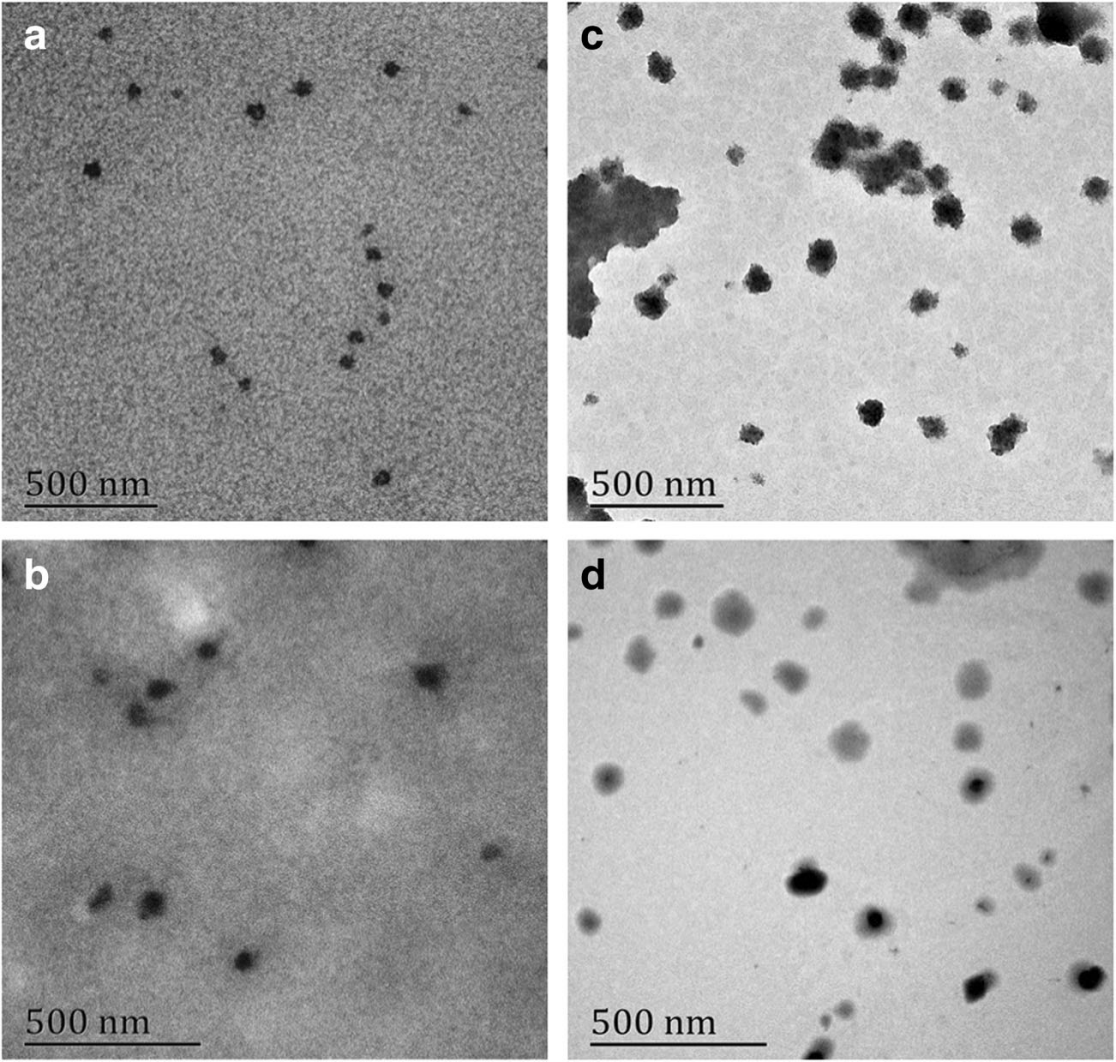
TEM images of PEG_113_-*b*-PDEA_50_-*b*-PGAMA_20_ block glycopolymer micelles before (**a**) and after BTZ-loading (**c**) at pH 9 and PEG_113_-*b*-PS_50_-*b*-PGAMA_20_ block glycopolymer micelles before (**b**) and after BTZ-loading (**d**) at pH 9

Those self-assembly micelles were measured also by TEM measurement, and TEM images of PEG_113_-*b*-PS_50_-*b*-PGAMA_20_ and PEG_113_-*b*- PDEA_50_-*b*-PGAMA_20_ triblock glycopolymers after self-assembly as shown in Fig. 7 revealed the presence of presumably spherical micelles of 40–50 nm and 50–60 nm in diameter at pH 9, respectively. The particle sizes estimated from TEM were systematically smaller than those obtained by DLS for the former reflects conformations in the dry state, while the latter reflects the swollen states at water media.

Therefore, PEG_113_-*b*-PS_50_-*b*-PGAMA_20_ triblock glycopolymer was formed stable micelles with PS core and PEG and PGAMA outer shell with diameter at 60 nm at any pHs, while PEG_113_-*b*-PDEA_50_-*b*-PGAMA_20_ triblock glycopolymer exhibited clear pH-responsive behaviors and self-assembled into micelles with diameter at 70 nm only at pH above 7, while dissociated to unimers at pH below 6. PEG_113_-*b*-PGAMA_20_ diblock glycopolymer keeps unimer state at whole pH ranges.

### Stability of the micelles formed by triblock glycopolymers

**Fig. 8:**
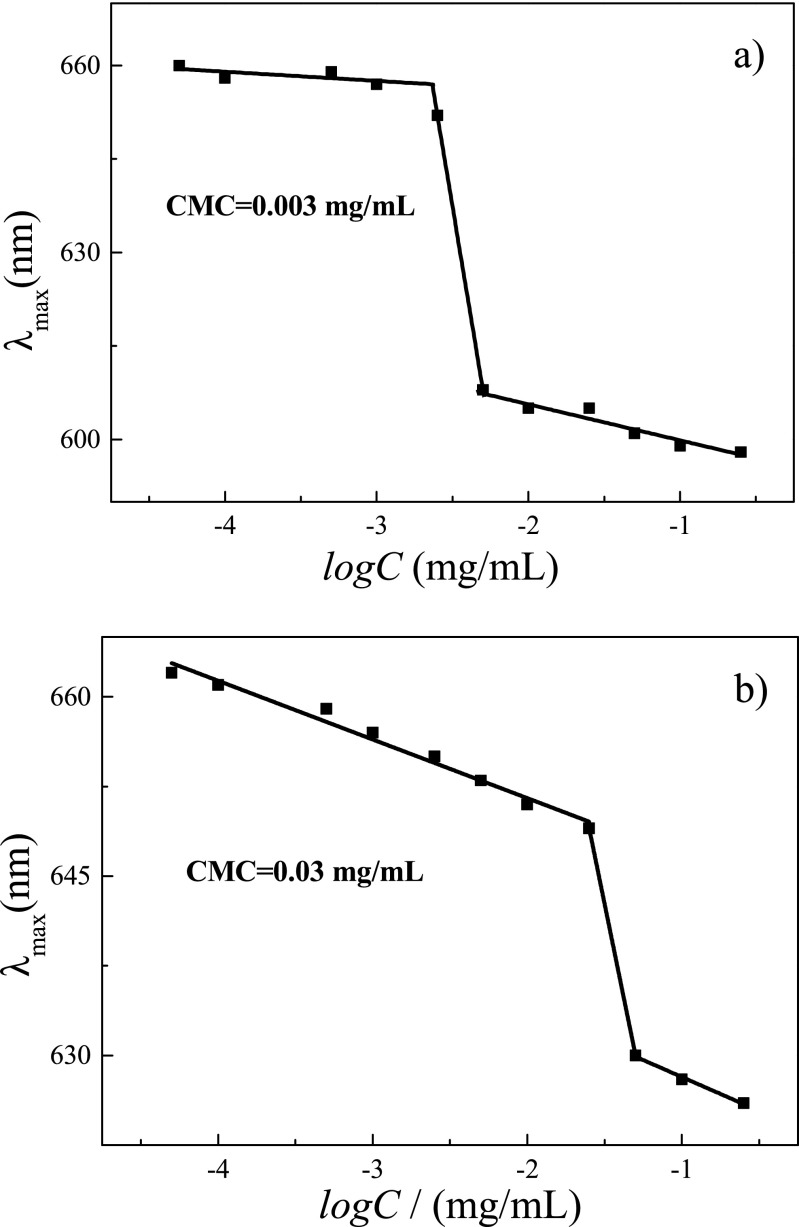
Determination of CMC with the dependence of maximum emission wavelength (*λ*_max_) of Nile Red on the logarithmic concentration of triblock glycopolymers PEG_113_-*b*-PS_50_-*b*-PGAMA_20_ (**a**) and PEG_113_-*b*-PDEA_50_-*b*-PGAMA_20_ (**b**) triblock glycopolymers at pH 9

Those triblock glycopolymers have exhibited self-assembly behaviors and would be used as drug delivery system; thus, critical micelle concentrations (CMC) of triblock glycopolymers were determined with Nile Red as probe by detecting the excitation maximum peak as collected in Fig. 8. For PEG_113_-*b*-PS_50_-*b*-PGAMA_20_ triblock glycopolymer, there is almost no change of excitation maximum peak (*λ*_max_) in fluorescence spectra at concentration of 0.003 mg/mL or less, while blue shift could be observed at solution concentration above 0.003 mg/mL. Thus the CMC of triblock glycopolymer PEG_113_-*b*-PS_50_-*b*-PGAMA_20_ could be calculated to be 0.003 mg/mL by plotting the λ_max_ of fluorescence spectra with logarithms of corresponding solution concentration, and then the CMC of PEG_113_-*b*-PDEA_50_-*b*-PGAMA_20_ triblock glycopolymer at pH 9 was also measured to be 0.03 mg/mL using similar method. The lower CMC value of PEG_113_-*b*-PS_50_-*b*-PGAMA_20_ triblock glycopolymer than that of PEG_113_-*b*-PDEA_50_-*b*-PGAMA_20_ demonstrates better stability and stronger self-assembly ability due to stronger hydrophobicity of PS block than deprotonated PDEA block, although both triblock glycopolymers possess the same DP of middle block.

### Bortezomib loaded micelles self-assembled from block glycopolymers

**Scheme 2:**
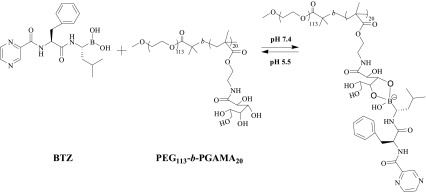
The pH-induced interaction of PEG_113_-*b*-PGAMA_20_ diblock glycopolymer with BTZ

The pH-induced dynamic covalent interactions between boronic acid and diols have been proofed at different systems including molecules or polymers containing boronic acid moiety with glycopolymers [43–45]. Therefore, the interaction of glycopolymers with boronic acid-containing BTZ at pH 7.4 was investigated for glycopolymers utilized as drug delivery systems of BTZ, and chromogenic reaction of Alizarin Red S (ARS) has been already used to detect this kind of interaction [46]. ARS is a catechol dye with burgundy color at PBS buffer solution (pH 7.4), and the color changes to yellow after addition of BTZ due to the boronic acid-catechol interaction, then the color of the mixture returns back to burgundy upon the addition of PEG_113_-*b*-PGAMA_20_ diblock glycopolymer, indicating the glycopolymers have stronger interaction with BTZ due to abundant hydroxyl groups, then release the free ARS from ARS-BTZ complex back to PBS solution. This phenomenon is consistent with that of boronic acid-containing molecules or copolymers with glycopolymers and glucose reported in other works [31], and their pH-induced interaction of PEG_113_-*b*-PGAMA_20_ diblock glycopolymer with BTZ was shown in Scheme 2.

**Fig. 9:**
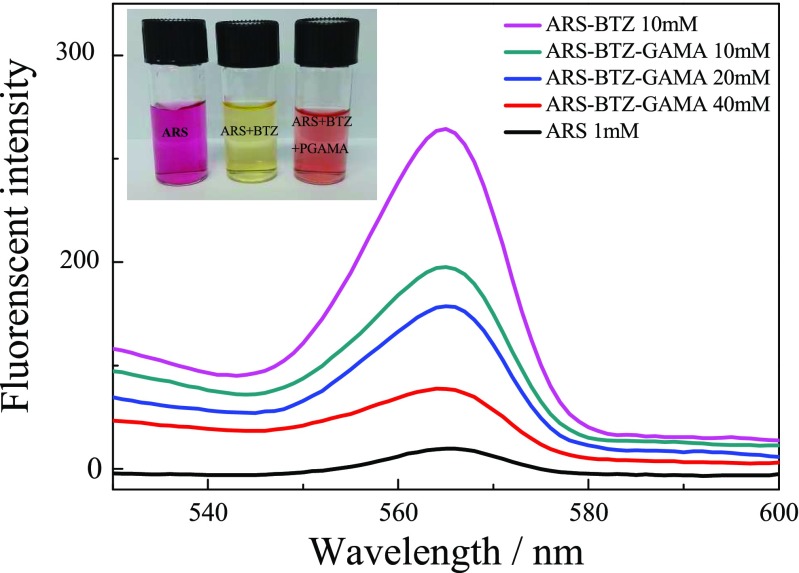
Fluorescence spectra and visual inspection of ARS, ARS and BTZ, and ARS and BTZ with various concentrations of PEG_113_-*b*-PGAMA_20_ diblock glycopolymer in PBS buffer (0.01 M, pH 7.4)

To further proof their interaction of glycopolymer with BTZ, the changes of their fluorescence spectra were recorded at PBS buffer solution (pH 7.4) as shown in Fig. 9. ARS has week fluorescence intensity, ARS also leads to dramatic changes in fluorescence intensity after forming esters with boronic acids, and the fluorescence spectra were recorded with various ratios of boronic acids and glucose moieties [46]. As the concentration of PEG_113_-*b*-PGAMA_20_ added increased, the fluorescence intensity of mixed solution of ARS and BTZ obviously decreased to near single ARS solution without fluorescence effect, which demonstrated the formation of boronic esters between BTZ containing boronic acids and glycopolymer as ARS was deterred from complex with BTZ.

Thus PEG_113_-*b*-PGAMA_20_ diblock glycopolymer could be utilized to load the BTZ at pH 7.4 via the glycopolymer-boronic acid interaction, then diblock glycopolymer was mixed with BTZ at DMSO then changed to water media at pH 7.4 to induce their interaction. After dialysis and lyophilization process, the loading capacity (LC) and encapsulation efficiency (EE) of drug BTZ by PEG_113_-*b*-PGAMA_20_ diblock glycopolymer carrier were detected to be 12.4 and 84.5%, respectively, indicating that the glycopolymers have the capacity of loading drugs containing boronic acid moiety via dynamic covalent bonding.

Following the same principle, two triblock glycopolymers, PEG_113_-*b*-PS_50_-*b*-PGAMA_20_ and PEG_113_-*b*-PDEA_50_-*b*-PGAMA_20_, were utilized also as delivery systems of BTZ, and their loading capacity (LC) and encapsulation efficiency (EE) of drug BTZ were detected to be 9.8 and 81% and 7.6 and 72%, respectively. The results show that the PEG_113_-*b*-PGAMA_20_ diblock glycopolymer has the highest LC and EE values, the reason is probably the diblock glycopolymer complexes via dynamic covalent bonding with BTZ in unimer state and forms the loose hydrophobic region, and meanwhile BTZ was physically encapsulated also into the glycopolymer carrier due to less steric hindrance effect by comparing with other triblock glycopolymers carriers, which possess already micellar structures with hydrophobic PS or PDEA cores and PEG block attached on outer shell of carriers.

The PEG_113_-*b*-PS_50_-*b*-PGAMA_20_ glycopolymer carrier has higher LC and EE than that of PEG_113_-*b*-PDEA_50_-*b*-PGAMA_20_ glycopolymer carrier due to the strong hydrophobic property of PS block, which have the stronger affinity with BTZ by low CMC than that of PDEA block via hydrophobic interaction, although those glycopolymers contain the same DPs of each block. Those results further indicate those glycopolymers carriers loaded BTZ not only via pH-induced dynamic interaction at pH 7.4, but the hydrophobic interactions also existed in those systems.

**Fig. 10:**
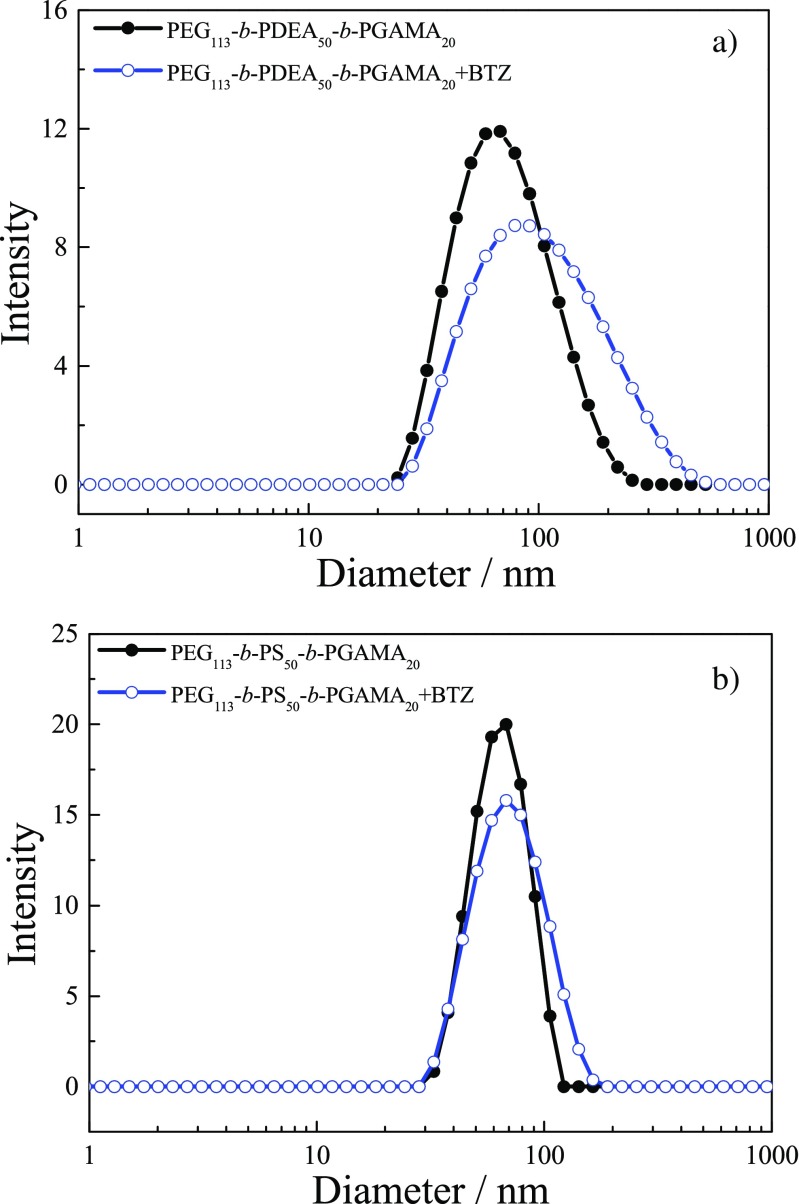
Intensity size distribution of PEG_113_-*b*-PDEA_50_-*b*-PGAMA_20_ (**a**) and PEG_113_-*b*-PS_50_-*b*-PGAMA_20_ (**b**) glycopolymer micelles before and after drug loading

After loaded BTZ, the triblock glycopolymer carriers in PBS solution at pH 7.4 were also studied by DLS (see Fig. 10). The diameters of PEG_113_-*b*-PS_50_-*b*- PGAMA_20_ and PEG_113_-*b*-PDEA_50_-*b*-PGAMA_20_ carriers were measured to be 60 nm and 70 nm, respectively. There is almost no change for PEG_113_-PS_50_-PGAMA_20_ triblock glycopolymer micelle before and after the loading of BTZ for the hard hydrophobic PS inner core; thus, the diameter size of PEG_113_-*b*-PDEA_50_-*b*-PGAMA_20_ carrier slightly reaches to around 80 nm after loading BTZ for the soft hydrophobic PDEA core. Those results showed that BTZ loading had no significant effect on glycopolymer micelle size. At the same time, the micelles retained the spherical morphology from their TEM images (Fig. 7) after loading BTZ, and their radius sizes were close to those of DLS measured.

### PH-dependent bortezomib release from block glycopolymers

**Fig. 11:**
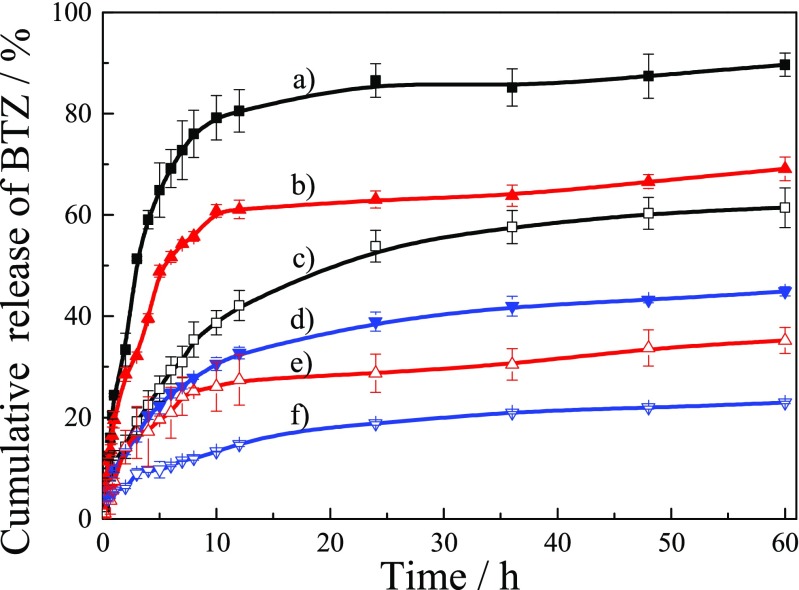
Time-dependent release of BTZ from PEG_113_-*b*-PGAMA_20_ diblock glycopolymer, PEG_113_-*b*-PDEA_50_-*b*-PGAMA_20_ triblock glycopolymer, and PEG_113_-*b*-PS_50_-*b*-PGAMA_20_ triblock glycopolymer at pH 5.5 (a, b, and d) and pH 7.4 (c, e, and f), respectively

Those block glycopolymer carriers were measured their release behaviors of BTZ by mimicking pH environments of the normal cell at pH 7.4 and tumor cell at 5.5 via conventional membrane dialysis method, and the released BTZ was detected by measuring their UV-visible absorption, then transferred to cumulative release concentration of BTZ, and all data were collected in Fig. 11.

For PEG_113_-*b*-PGAMA_20_ copolymer carrier, around 40% of the payload was released within 10 h and increased to be 60% within 60 h at pH 7.4, this is probably part of BTZ escaping from carrier was physically attached glycopolymer carrier system via week hydrophobic interaction. Then at pH 5.5, about 80% of the payload was released within 10 h and up to 90% within 60 h, which is from the structural dissociation of glycopolymer carrier system with BTZ and then BTZ was burst released from carrier system due to the broken of pH-induced dynamic covalent bonding between the glycopolymer and BTZ.

For PEG_113_-*b*-PS_50_-*b*-PGAMA_20_ triblock copolymer carrier, about 13% of the payload was released within 10 h and slightly increased to be 20% over 60 h at pH 7.4. The structure of micelles with PS cores is very stable and the glycopolymer carrier keeps the interaction with BTZ via dynamic covalent bonding; thus, drug have strong interaction with glycopolymer carrier so that BTZ was hard to be released. At pH 5.5, the amount of released payload had a moderate increase to be 30% within 60 h, because BTZ conjugated with PGAMA block was fast released due to breakage of borate ester bonds in response to acid environment whereas BTZ aggregated in core of micelles was not easy to escape from interaction with hydrophobic PS block [47, 48].

Drug-releasing rate of PEG_113_-*b*-PDEA_50_-*b*-PGAMA_20_ glycopolymer carrier was also slow at pH 7.4 with 20% within 10 h. This suggested that the complexation between BTZ and glucose groups is robust at pH 7.4, and the stability of micelles could also effectively prevent the burst release of BTZ at physiological environment (pH 7.4) before carried to lesions (nearly pH 5.5). At pH 5.5, about 60% of the payload was released within 10 h ascribed to synergistic effects of PDEA block deprotonated to be hydrophilic causing dissociation of micelles and chemical decomplexation of BTZ with PGAMA block under the stimulus of acid pH, and this double fast pH-responsiveness led to rapid release of BTZ [49, 50]. It should be noted that the amount of drug release for PEG_113_-*b*-PDEA_50_-*b*-PGAMA_20_ glycopolymer carrier was not more than 70% within 60 h at pH 5.5, showing appreciable sustained release effect. Those three glycopolymers were identified to realize the BTZ release via a controlled manner and then could be utilized to meet different demands in BTZ delivery systems.

The cumulative release curves of PEG-*b*-PDEA-*b*-PGAMA and PEG-*b*-PGAMA carriers at pH 7.4 and 5.5 were measured to figure out the pH-induced release of those two carries with or without pH-responsive cores. It was supposed that the PEG-*b*-PDEA-*b*-PGAMA triblock glycopolymer carrier should exhibit the same release behaviors of BTZ at pH 5.5 with that of PEG-*b*-PGAMA carrier after long time interval, for both block glycopolymers were dissociated to unimers at acidic pH value. But it should be noted that PEG-*b*-PDEA-*b*-PGAMA glycopolymer carrier has the core-shell morphology with PDEA cores and PGAMA and PEG-mixed shells and the BTZ was mainly loaded at core of micelles and partially complexed with PGAMA outer layer, which is different with that of PEG-*b*-PGAMA carrier with PGAMA/BTZ aggregate cores and PEG shells and BTZ was mainly located at aggregate core. When pH value shifts from 7.4 to 5.5, two steps happened at the PEG-*b*-PGAMA carrier system: hydrogen ions pass the one layer of PEG shell and then enter the aggregate core to induce the de-complexation of BTZ with PGAMA and dissociate the aggregate to release BTZ drug. While, hydrogen ions have to pass the mixed layer of PEG and PGAMA shell to break the interaction of PGAMA/BTZ, enter the aggregate core to induce the dissociation of micelles, and then BTZ drug was released at the PEG-*b*-PDEA-*b*-PGAMA carrier system. Two pH-induced dissociation processes, de-complexation of BTZ with PGAMA at mixed shell and protonation of PDEA at core of micelles, happened and both processes resulted in the slower release velocity and lower release content of BTZ of PEG-*b*-PDEA-*b*-PGAMA glycopolymer carrier system than those of PEG-*b*-PGAMA carrier system.

## Conclusions

Three block glycopolymers, PEG_113_-*b*-PGAMA_20_, PEG_113_-*b*-PS_50_-*b*-PGAMA_20_, and PEG_113_-*b*-PDEA_50_-*b*-PGAMA_20_, were prepared via ATRP technique and utilized as drug delivery system to investigate the loading and release of boronic acid-containing bortezomib (BTZ). Those glycopolymers were demonstrated to possess the capacity of loading anticancer drug BTZ at physiological pH 7.4 via conjugation method by the dynamical covalent complexation between glucose and boronic acid, which made BTZ attached to shell of micelles, and physical encapsulation method by accumulating in the core of micelles due to hydrophobicity of drugs. PEG_113_-*b*-PS_50_-*b*-PGAMA_20_ triblock copolymer carrier had good ability to load bortezomib both at pH 7.4 and at pH 5.5 (nearly acid environment of cancer cells) with non-responsive PS block supporting stable structure of micelles to gather BTZ. PEG_113_-*b*-PDEA_50_-*b*-PGAMA_20_ carrier with pH-responsive core exhibited better controlled and sustained release of BTZ attributed to the synergistic pH stimulating responsiveness from the breakage of boronate esters and dissociation of micelles at pH 5.5. Those glycopolymers equipped with ability to self-assembly into micelles provide a facile and promising polymeric nanocarrier system for bortezomib also with further great design flexibility aiming to minimize the premature release of drug and accomplish accurate controlled release in vivo.
